# Deep Learning Radiomics Nomogram to Predict Lung Metastasis in Soft-Tissue Sarcoma: A Multi-Center Study

**DOI:** 10.3389/fonc.2022.897676

**Published:** 2022-06-24

**Authors:** Hao-yu Liang, Shi-feng Yang, Hong-mei Zou, Feng Hou, Li-sha Duan, Chen-cui Huang, Jing-xu Xu, Shun-li Liu, Da-peng Hao, He-xiang Wang

**Affiliations:** ^1^Department of Radiology, The Affiliated Hospital of Qingdao University, Qingdao, China; ^2^Department of Radiology, Shandong Provincial Hospital Affiliated to Shandong First Medical University, Jinan, China; ^3^Department of Radiology, The Third People’s Hospital of Qingdao, Qingdao, China; ^4^Department of Pathology, The Affiliated Hospital of Qingdao University, Qingdao, China; ^5^Department of Radiology, The Third Hospital of Hebei Medical University, Shijiazhuang, China; ^6^Department of Research Collaboration, Research and Development (R&D) Center, Beijing Deepwise & League of Philosophy Doctor (PHD) Technology Co., Ltd, Beijing, China

**Keywords:** magnetic resonance imaging, deep learning, radiomics nomogram, soft tissue sarcomas, lung metastasis

## Abstract

**Objectives:**

To build and evaluate a deep learning radiomics nomogram (DLRN) for preoperative prediction of lung metastasis (LM) status in patients with soft tissue sarcoma (STS).

**Methods:**

In total, 242 patients with STS (training set, n=116; external validation set, n=126) who underwent magnetic resonance imaging were retrospectively enrolled in this study. We identified independent predictors for LM-status and evaluated their performance. The minimum redundancy maximum relevance (mRMR) method and least absolute shrinkage and selection operator (LASSO) algorithm were adopted to screen radiomics features. Logistic regression, decision tree, random forest, support vector machine (SVM), and adaptive boosting classifiers were compared for their ability to predict LM. To overcome the imbalanced distribution of the LM data, we retrained each machine-learning classifier using the synthetic minority over-sampling technique (SMOTE). A DLRN combining the independent clinical predictors with the best performing radiomics prediction signature (mRMR+LASSO+SVM+SMOTE) was established. Area under the receiver operating characteristics curve (AUC), calibration curves, and decision curve analysis (DCA) were used to assess the performance and clinical applicability of the models.

**Result:**

Comparisons of the AUC values applied to the external validation set revealed that the DLRN model (AUC=0.833) showed better prediction performance than the clinical model (AUC=0.664) and radiomics model (AUC=0.799). The calibration curves indicated good calibration efficiency and the DCA showed the DLRN model to have greater clinical applicability than the other two models.

**Conclusion:**

The DLRN was shown to be an accurate and efficient tool for LM-status prediction in STS.

## Introduction

Soft tissue sarcomas (STS) are rare malignant neoplasms having unpredictable clinical and pathologic behaviors (1). Approximately 25%–30% of patients with STS have distant metastasis (DM), which is associated with a poor prognosis (2, 3), with this DM rate rising to 50% in high-grade STS (4). The lung is the most common site of DM (5),with approximately 80% of DM cases occurring in STSs of the extremities (6). When complete lesion resection of pulmonary metastases can be made, the 3-year survival rates of patients with metastasizing STS can reach 30%–46% (7–9). Thus, with the risk of lung metastases (LM) from STS, there is a need to supply systemic therapy at the earliest possible time (10). In this condition, more aggressive chemotherapy or cancer treatment targeted to the histopathology of the STS could be carried out (11, 12). The accurate and early identification of LM risks in the period of STS therapy is thus of central importance because it could potentially indicate the most appropriate treatment and enhance overall survival.

The most common appearance of LMs of STSs is as a pulmonary nodule. However, chest computed tomography (CT) cannot effectively differentiate metastatic lung nodules from non-metastatic ones, and positron emission tomography (PET)-CT scan supplies few extra clinical benefits because of its high false-negative rate for lung nodules ≤ 10 mm in diameter (13).

Radiomics is a promising prospect that involves the extraction of large numbers of high-throughput analysis features from medical images, and can consequently be used to screen for vital features for use in models for quantitative oncology diagnostics (14, 15). Although radiomics can quantitatively represent intra-tumoral heterogeneity (14), partial volume effects may mean that the heterogeneity of small lesions may not be accurately quantified (16). Hatt et al. (17) suggested that for tumors < 10 cm^3^, radiomics texture features have no additive value in outcome forecasting. Most early LM lesions are usually small and may therefore not be suitable for radiomics analysis; for a single pulmonary nodule, puncture biopsy is undoubtedly a more appropriate strategy. Nevertheless, when multiple lung nodules (including metastatic and non-metastatic nodules) are present, false negatives can sometimes occur because of sample selection. In summary, analysis of lung nodules cannot always effectively identify the LM status of STS.

Past studies have suggested that tumor-related risk factors, such as the grade of malignancy and size of the tumor, are prognostic factors for the DM status of STS (18, 19). Although magnetic resonance imaging (MRI) is indispensable for the routine management of patients with STS, conventional imaging assessment relying on the manual evaluation of semantic features of masses by expert radiologists can suffer from a relative paucity of features, and it neglects a large amount of information on tumor heterogeneity (20). Radiomics uses analyzable image information to improve the clinical decision strategy, and can enhance the performance of oncology diagnosis and prognosis (21). Deep learning (DL), which involves convolutional neural networks, has frequently been applied to radiological imaging features and has shown very good performance in cancer prognosis (22). At least two radiomics models using primary lesion evaluation to predict DM-status in STS have been described (23, 24), and a DL model using PET and MRI texture features of the primary lesion was constructed to predict the LM-status in STS (12). However, a model based on MRI handcrafted radiomics (HCR) and DL features to predict LM-status in STS has not yet been reported.

The purpose of our study was therefore to construct a DL radiomics nomogram (DLRN) using a three-center dataset for the preoperative prediction of LM status in STS.

## Materials and Methods

### Patients and Tumor Characteristics

All organizations that participated in this retrospective study achieved approval from their hospital ethics review board and a waiver for the provision of written consent. A total of 351 patients were retrospectively identified for enrollment into this study. All patients underwent preoperative MRI and chest CT examinations at one of three hospitals and were confirmed as having STS by postoperative pathology between May 2008 and September 2020. **Supplementary Item A1** lists the inclusion and exclusion criteria applied to the patients. Finally, 242 patients (median age 53, from 1 to 93) were enrolled in the current study, and were divided into a training set of 116 patients from Institution 1 (Affiliated Hospital of Qingdao University, Qingdao, China) and an external validation set consisting of 126 patients from Institutions 2 and 3 (Institution 2: Shandong Provincial Hospital Affiliated to Shandong First Medical University, Jinan, China; Institution 3: The Third Hospital of Hebei Medical University, Shijiazhuang, China). Preoperative clinical information, including age, gender, TNM stage, and semantic MRI features, was obtained. The TNM staging was evaluated using the preoperative MRI and CT according to the American Joint Committee on Cancer (AJCC) Staging Manual (8^th^ Edition). Pathological results were confirmed by a pathologist (F.H.) with 12 years of experience in soft tissue disease diagnosis. The final histopathologic results of the 242 STS patients are shown in **Table S1**. LM was confirmed by continued progression of the pulmonary nodule on regular postoperative CT examinations or by histopathological diagnosis of puncture or surgical resection samples.

### MRI and Semantic Feature Acquisition

All patients underwent preoperative MRI examinations, including axial T1-weighted imaging (T1WI) and axial fat-suppressed T2-weighted imaging (FS-T2WI). **Supplementary Item A2** lists the MRI acquisition settings, and the analysis of the MRI semantic features is summarized in **Supplementary Item A3**.

### Region-of-Interest Delineation and Radiomics Feature Extraction

**Figure 1:**
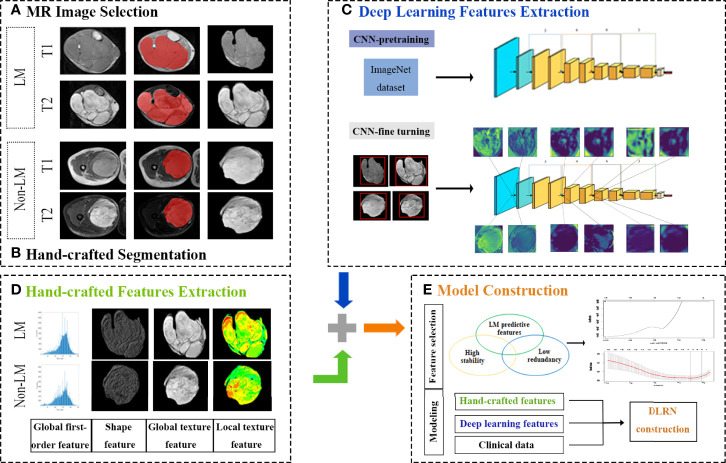
Schematic of the radiomics analysis.

A schematic of the radiomics analysis is shown in **Figure 1**. Region-of-interest (ROI) segmentation in three dimensions was applied with ITK-SNAP software (version. 3.8.0, http://www.itksnap.org). A total of 1379 HCR features were derived from each ROI (the extraction flow is shown in **Supplementary Item A4**).

For the deep leaning-based feature extraction process, we used the deep convolution network ResNet34 as DL feature extractor (25). The model was pre-trained on Image-Net dataset based on Pytorch 1.4.0 platform for transfer learning. The output of the last convolutional layer of ResNet-34 were used as DL features. After elimination of null features, 104 deep leaning-based features (54 from T1WI and 50 from FS-T2WI) were forwarded into the following processes.

### Combat Compensation Methodology

The combat compensation methodology (26) was used to remove the effects of different protocols and scanners, with the method filtering out technical inconsistencies in MRI radiomics features (27). In this study, combat was applied to decrease the inconsistencies of multi-central radiomics features.

### Handcrafted Radiomics and Deep Learning Signature Building

After the combat compensation method, all hand-crafted radiomics features were standardized to z-score. The feature selecting method of minimum redundancy maximum relevance (mRMR) was applied to select the top 15 features. Then, the least absolute shrinkage and selection operator (LASSO) algorithm was used to further screen the feature parameters. The LASSO algorithm compresses the regression coefficients of some features to zero, and a 10-fold cross verification method was applied to select the best weight coefficient λ. The selected features were combined with their respective coefficients using a linear combination formula to form the radiomics model. Five machine-learning classifiers were compared: logistic regression, decision tree, random forest, support vector machine (SVM), and adaptive boosting; and the method with the best prediction performance was used to construct a predictive radiomics signature.

Finally, the following prediction models were built: (1) an HCR model, including T1WI and FS-T2WI handcrafted radiomics features; (2) a DL model, including T1WI and FS-T2WI DL extracted features; and (3) a handcrafted and radiomics-DL combined (HD-Combined) model, including both HCR features and DL features from T1WI and FS-T2WI. Each machine-learning model was first trained without subsampling and then retrained with the synthetic minority oversampling technique (SMOTE) (28, 29).

### Clinical Model and Building of the Deep Learning Radiomics Nomogram

The statistically significant preoperative clinical characteristics were screened using univariate logistic analysis. Characteristics with P < 0.10 were then entered into a multivariate logistic regression. Those characteristics with a P value < 0.05 were identified as independent predictors for a risk of LM and were used to establish a preoperative clinical model. Finally, the independent risk predictors and the best performing radiomics signature model were combined to construct the DLRN.

### Performance Assessment of the Deep Learning Radiomics Nomogram and Different Models

The area under the receiver operating characteristics curve (AUC) and accuracy were used to access the LM-status prediction capability of the clinical model, radiomics signature models, and DLRN when they were applied to the training set and external validation set. The DeLong test was performed to evaluate the difference of each model’s AUC. Calibration curves were used to assess the fitting of the models. The clinical reliability and practicability of the models were evaluated by decision curve analysis (DCA).

### Follow-up Surveillance and Survival Analysis

Postoperative follow-up examinations of the patients using MRI or CT were performed every 3–6 months for the first 2 years and then once a year subsequently. The time from operation to the time of the patient survival endpoint outcome, such as imaging examination identification of lesion recurrence, day of last follow-up examination, or day of death with no evidence of progression, was counted as progression-free-survival (PFS). The censoring criteria for the patients were no matter emigration, or the 31 October 2020, whichever came first.

Kaplan-Meier survival curves were used for PFS analysis, and the log-rank test was used to analyze the survival situation and compare the PFS probability of patients in different metastasis risk groups. The DLRN model was enrolled into the PFS stratification evaluation.

### Statistics

All statistical procedures were performed using R software (v 3.4.4, http://www.r-project.org) and R packages we used in each step were shown in **Table S2**. All p-values of < 0.05 were considered statistically significant. Student’s *t*-test was used for continuous variables and the chi-square test for class-based variables. Uni- and multi-variate logistic analysis were applied in SPSS software (IBM, v 25.0).

## Results

### Clinical Information Screening and Model Construction

The preoperative clinical information and semantic MRI features of the 242 patients with STS are shown in **Table 1**. The univariate and multivariate logistic regression results are shown in **Table 2**. According to the results of the univariate logistic regression analysis, four clinical parameters showed a significant contribution to the prediction of the LM-status of patients with STS. However, following the multivariate logistic regression, only one clinical parameter (T-stage) was included in the clinical model construction. The AUC values of the clinical model were 0.696 on the training set and 0.664 on the external validation set (**Table 3**).

**Table 1:** Patient’s Clinical information and MRI semantic features between non-metastasis and metastasis group in the training and external validation set.

		Training set (N = 116)			External validation set (N = 126)		
		Non-metastasis(N = 96)	Metastasis(N = 20)	P	Non-metastasis (N = 107)	Metastasis (N = 19)	P
Age (years) (mean ± SD)		51.31 ± 18.851	48.10 ± 17.741	0.485	51.51 ± 17.159	49.74 ± 20.448	0.687
Gender	Male	62	12	0.698	63	15	0.097
	Female	34	8		44	4	
T-stage	1	33	3	0.002	28	3	0.022
	2	44	7		55	6	
	3	11	2		17	5	
	4	8	8		7	5	
N-stage	0	86	15	0.161	98	14	0.022
	1	10	5		9	5	
MRI Semantic Features							
Number	Solitary	62	16	0.181	76	15	0.478
	Multiple	34	4		31	4	
Depth	Deep	37	10	0.344	56	55	0.036
	Superficial	59	10		51	14	
Heterogeneous SI at T1WI	<50%	62	8	0.041	53	9	0.862
	≥50%	34	12		54	10	
Heterogeneous SI at T2WI	<50%	50	6	0.072	48	7	0.516
	≥50%	46	14		59	12	
Tumor volume of necrosis MRI signal	0	20	5	0.590	22	1	0.265
	1%–50%	55	9		60	12	
	>50% of tumor volume	21	6		25	6	
Peritumoral edema	No	20	5	0.178	28	4	0.045
	Limited	62	9		71	10	
	Extensive	14	6		8	5	
Location	Limb	93	19	0.648	61	9	0.202
	Trunk wall	2	1		16	4	
	Head and neck	0	0		2	2	
	Internal trunk	1	0		28	4	

SD, standard deviation; Calculated from student t-test or Mann–Whitney U test for ordinal variables and chi-square test or Fisher exact test for categorical variables, where appropriate.

**Table 2:** Results of univariate and multivariate logistic regression analysis in soft-tissue sarcoma patients.

	Univariate Logistic Analysis		Multivariate Logistic Analysis	
	OR (95%CI)	P	OR (95%CI)	P
**Radi-socre**	**5.540 (2.814-10.907)**	**<0.001**	**6.617 (2.755-15.891)**	**<0.001**
Age	0.991 (0.966-1.017)	0.482		
Gender	0.823 (0.306-2.208)	0.698		
**T-stage**	2.201 (1.351-3.583)	0.002	2.943 (1.266-6.838)	0.012
**N-stage**	0.349 (0.104-1.165)	0.087	0.153 (0.023-1.007)	0.051
Number	0.456 (0.141-1.473)	0.189		
Depth	0.627 (0.238-1.651)	0.345		
**Heterogeneous SI at T1WI**	0.366 (0.136-0.981)	0.046	1.211 (0.126-11.677)	0.869
**Heterogeneous SI at FS-T2WI**	0.394 (0.140-1.112)	0.079	1.284 (0.131-12.577)	0.830
Tumor volume with MRI signal compatible with necrosis	1.092 (0.531-2.246)	0.810		
Peritumoral edema	1.340 (0.614-2.922)	0.462		
Location	0.979 (0.209-4.582)	0.979		

OR, odds ratio; CI, confidence interval.

**Table 3:** Results of clinical model, radiomics model and DLRN predictive performance.

Set	Model	AUC (95%CI)	ACC	ER	SEN	SPE	PPV	NPV	P
Training	DLRN	0.936 (0.874-0.999)	0.914	0.086	0.650	0.969	0.813	0.930	Reference
	Radiomics model	0.914 (0.876-0.953)	0.755	0.245	0.463	1.000	1.000	0.691	0.551
	Clinical model	0.696 (0.564-0.827)	0.828	0.172	0.000	1.000	NA	0.828	<0.001
External validation	DLRN	0.833 (0.732-0.933)	0.897	0.103	0.474	0.972	0.750	0.912	Reference
	Radiomics model	0.799 (0.675-0.922)	0.881	0.119	0.263	0.991	0.833	0.883	0.394
	Clinical model	0.664 (0.523-0.805)	0.849	0.151	0.000	1.000	NA	0.849	0.034

CI, confidence interval; ACC, accuracy; ER, error rate; SEN, sensitivity; SPE, specificity; PPV, positive predictive value; NPV, negative predictive value; NA, not available.

### Feature Screening and Performance of the Radiomics Signatures

**Figure 2:**
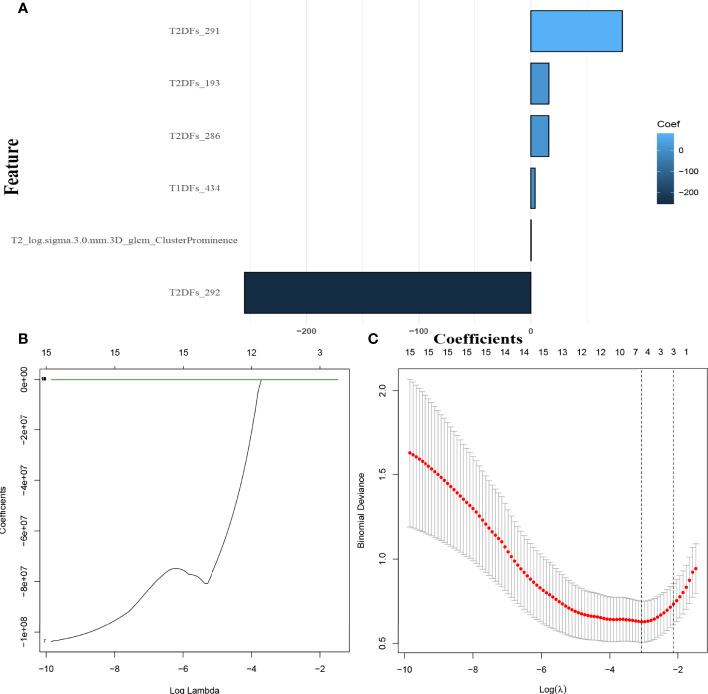
Selection of MRI hand-crafted radiomics and deep learning features. **(A)** The six radiomics features with non-zero coefficients in the HD-Combined model. **(B)** The coefficients plot (as ln λ). **(C)** Selection of the tuning parameter (λ). λ=0.057(ln λ=-2.86) was applied.

Overall, 949 HCR features from T1WI and 772 from FS-T2WI showed high stability (ICC > 0.75), and these were combined with 54 T1WI-DL features and 50 FS-T2WI-DL features for inclusion in the subsequent investigations. In the screening with the mRMR-LASSO algorithm, one HCR feature and five DL features had non-zero coefficients and were used in the HD-Combined model (**Figures 2A–C**). The features included in each model are shown in **Table S3**, while **Table S4** shows the predictive performance of all radiomics signatures. The HD-Combined signature trained by the SVM classifier had the best performance, with AUC of 0.806 and accuracy of 0.849 on the external validation set. **Table S5** shows the performance of all radiomics signatures combined with the SMOTE algorithm. The HD-Combined signature trained by the SVM classifier combined with the SMOTE algorithm attained the best performance, with AUC of 0.799 and accuracy of 0.881 on the external validation set. The HD-Combined SVM-SMOTE signature was selected to construct the DLRN because it gave the best accuracy for LM prediction in the external validation set.

### Validation of the Deep Learning Radiomics Nomogram and Patient Risk Stratification

**Figure 3:**
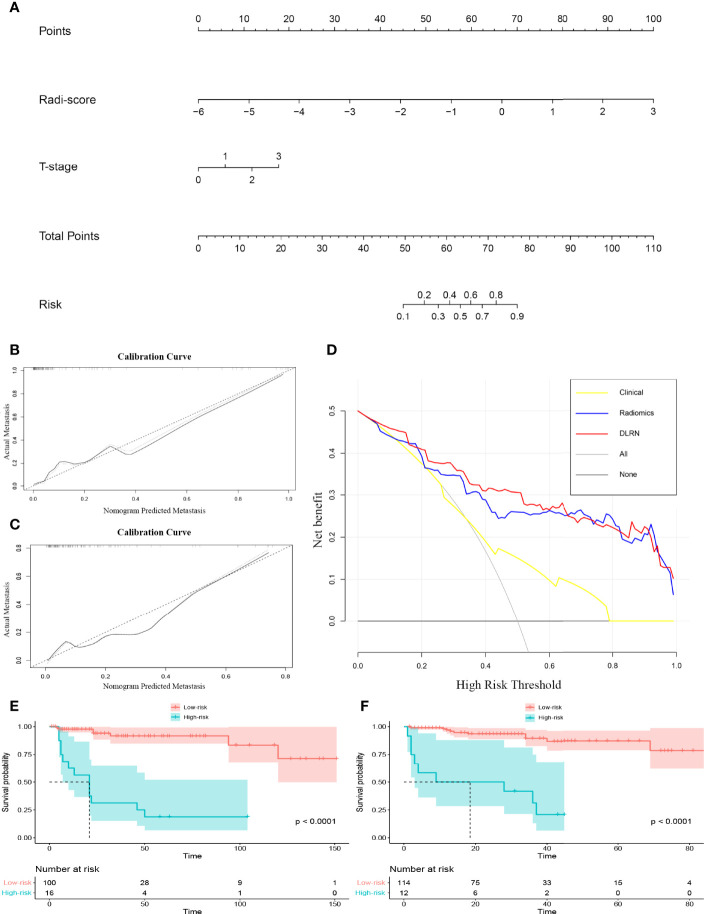
**(A)** Deep learning radiomic nomogram (DLRN). **(B)** Calibration curve of the DLRN on the training set. **(C)** Calibration curve of the DLRN on the external validation set. **(D)** Decision curve analysis of the DLRN. **(E, F)** Kaplan-Meier survival analysis of the DLRN model on the training and external validation sets.

A DLRN was constructed combining the HD-Combined SVM-SMOTE signature with the independent preoperative clinical LM predictor (**Figure 3A**), and **Table 3** shows its predictive performance. The AUCs showed significant difference between DLRN and clinical model (0.833 vs. 0.664, P < 0.05) on the external validation set. The AUCs were not significant different between DLRN and radiomics model (0.833 vs. 0.799, P = 0.394). The accuracy value of DLRN (0.897) is higher than that of clinical model (0.849) and radiomics model (0.881). **Figures 3B, C** shows the calibration curves of the DLRN, indicating that the DLRN was appropriate in both data sets. The DCA of the DLRN indicated it had better usefulness than the other two models (**Figure 3D**). As shown in the Kaplan–Meier survival curve, the DLRN model significantly stratified patients according to PFS in both the training and external validation sets (both log rank P < 0.01; **Figures 3E, F**). The median PFS times of low-LM-risk subsets were not reached in either of the sets, and the median PFS times of high-LM-risk subsets were 21.0 months in the training set and 18.5 months in the external validation set.

## Discussion

The objective of the current study was to build and validate LM risk prediction models based on MRI radiomics measured from the primary STS lesion. We found that in the LM-status prediction, the DLRN model of STS showed enhanced performance compared with the radiomics signatures and the clinical model alone, suggesting the incremental value of the DLRN to the current diagnostic management of STS. In this multi-institution study, the DLRN offered preferable prognostic ability (AUC=0.936 and 0.833, accuracy=0.914 and 0.897, for training and external validation sets, respectively), better clinical application, and was well calibrated. Additionally, the DLRN provided satisfactory PFS risk stratification of the patients.

In a previous study, Li (30) et al. found that certain clinical features, including age, histological subtype, primary location, tumor size, grade, and depth of extremities in STS, could be used as predictors of DM. Different patients may have different metastatic potential because of STS cell heterogeneity. In our study, our semantics-based clinical model showed poor differentiating performance, suggesting the limited value of visual MRI features and preoperative clinical data for prediction of LM in STS.

Radiomics is a promising approach that involves the extraction of large numbers of high-throughput analysis features, consequently allowing screening for vital features for use in models for quantitative oncology diagnostics (31, 32). In this study, the HD-Combined model showed good prediction performance that was significantly higher than the semantics-based clinical model on both training and external validation sets. This indicates that visual features reflect relatively little of the information present in images and neglect a large amount of information on tumor heterogeneity (14). We found that our HCR feature-based predictive model showed unconvincing performance in the differentiation, performing similar to the clinical model on the external validation set. A possible reason for this was our ROI selection; in this study, the HCR features were extracted from the primary tumor region rather than the peritumoral region. Past studies revealed that peritumoral radiomics features can be vital imaging biomarkers for tumor metastasis prediction (33). Therefore, further research is necessary to investigate whether peritumoral HCR feature-based radiomics could enhance the prediction model performance. The DL-feature-based model showed improved performance over the HCR model according to both AUC and accuracy. There might be two reasons for this. First, DL algorithms are able to create their own features for the classification (34). Radiomics models combined with DL features showed good performance in tumor prognosis evaluation (22). Second, our DL models included DL-features extracted from peritumoral regions, which have been identified as vital areas for cancer prognosis prediction (35).

The best-performing HD-Combined model included 1 HCR feature and 5 DL features, suggesting that the DCNNs may have derived quantitative information reflecting the risk of LM occurring in STS. As displayed in **Figure S1**, the DCNN activation maps highlighted certain parts of the tumors with high predictive value for LM status, with these regions being suppressed in tumor with lower values. We deduce that the highlighted regions in the activation maps may have greater association with cancer metastasis. The adopted HCR feature of “gray-level co-occurrence matrix Cluster_Prominence” can quantify the skewness and asymmetry of gray-level variability in the tumor ROI, which may be unrecognized by the naked eye. A previous study suggested that gray-level co-occurrence matrix features possessed energetic capacity to predict tumor metastasis status and had an indispensable role in radiomic signature construction (36).

In terms of simplicity and efficiency, we combined the mRMR and LASSO feature screening with five classifiers to establish the machine learning algorithm. The mRMR method is a novel feature screening algorithm that can screen radiomics features with more credible coefficients and fewer redundancies (37). LASSO is an algorithm generally applied to data with high feature dimensions to reduce the number of features and avoid over-fitting in the model construction process (38). SVM is a practical machine learning classifier with convincing generalization abilities for non-visual features (39). Combining all these capacities, the mRMR-LASSO regression algorithm integrated with the SVM classifier achieved the best prediction performance in the MRI-radiomics analysis.

LM masses were present in less than 20% of the patients with STSs, and therefore a data unbalance problem cannot be ignored. An unbalanced sets problem can potentially generate a negative effect on the application of machine-learning classification approaches (40, 41), and can be solved by using state-of-the-art subsampling techniques that synthesize new data points in the minority subset, which are regard as “suitable” policies in machine learning (41). The performance of the HD-Combined signature showed slightly enhanced accuracy (from 0.849 to 0.881 in the external validation set) when combined with the SMOTE algorithms, and gave results similar to those in a previous study (40).

DM occurs in approximately 25%–30% of patients with STS (2, 3), leading to a poor prognosis. Approximately 80% of DM cases in STS occur in the lung (6). In our study, lung nodules occurred in 32% (78/242) of patients and 50% (39/78) of them were LMs. Combining the follow up and post-operative pathological data, among all these LM cases, only 33% (13/39) of them were confirmed as single LM, and 66% (26/39) of them were multiple ones. For a single suspected LM nodule, puncture biopsy is undoubtedly an appropriate strategy. However, when multiple suspected LM nodules exist, false negatives caused by sample selection can occasionally occur. Thus, when multiple suspected LM nodules are present, preoperative recognition of true LM lesions is clinically important. Our DLRN gave a prognostic accuracy of 0.897 on the external validation set. Therefore, for a patient with a high risk of LM, selection as an operative candidate is necessary because complete resection of LM lesions can enhance survival time (7–9). The clinical use of this DLRN could not only prevent unnecessary surgery but also reduce the cost burden from regular postoperative examinations and the fear associated with false-positive diagnoses.

Another discovery was that the current DLRN showed satisfactory risk stratification performance for the PFS of patients in the training and external validation sets, which reflects the reality that STS patients with LM have a poor prognosis (2, 3). Past studies have suggested that radiomics features can be used as predictors of survival outcomes in patients with STS. Spraker et al. (42) found that an STS radiomics model could predict overall survival, and Peeken et al. (43) found that an FS-T2WI-based radiomics model achieved good prognostic performance in overall survival risk stratification. In our study, we established a DLRN model to evaluate survival prediction and showed convincing stratification of patients according to PFS. This model therefore has promising prospects in the long-term management of patients with STS and a high risk of LM.

The current study had several limitations. First, selection bias may occur whenever strict criteria are applied. Second, our study only contained patients from China. STS can have different biology and etiology in different races or countries; how this would affect our nomogram is unknown. Third, because of the small number of patients who underwent contrast-enhanced MRI scans, contrast-enhanced images were not included in our study. Further MRI sequences, such as dynamic contrast-enhanced MRI, diffusion-weighted imaging, diffusion kurtosis imaging, and intravoxel incoherent motion, could be collected and included in future studies to improve the model. Finally, although pleasing external validation results were acquired in our study, a large number of Gordian techniques in radiomics flow (e.g. automated segmentation, progressive isotropic interpolation, and stable feature screening) need to be enrolled in further studies to enhance the robustness and generalization of the radiomics model.

In conclusion, the current DLRN had good performance for predicting LM status in STS, and could offer essential information for formulating the treatment strategy.

## Data Availability Statement

The raw data supporting the conclusions of this article will be made available by the authors, without undue reservation.

## Author Contributions

H-YL and S-FY have contributed equally to this work. D-PH and H-XW conceptualized the study. H-MZ, H-XW, and D-PH contributed to the methodology and validated the study. S-LL, S-FY and L-SD organized the database. H-YL, C-CH and J-XX contributed to the formal analysis and the statistical analysis. H-YL and S-FY wrote and prepared the original draft. All authors wrote and prepared the original draft. D-PH, H-XW and S-FY supervised the study. All authors contributed to the article and approved the submitted version.

## Funding

This work was supported by the National Natural Science Foundation of China (Grant No. 82172035). This study was funded by the Project Grant No. ZR2020MH286 and No. ZR2021MH159 supported by the Shandong Provincial Natural Science Foundation. This study was funded by the Clinical Medicine +X Project of the Affiliated Hospital of Qingdao University (Grant No. QDFY+ X2021015). This work was supported by the Medicine and Health Technology Development Program of Shandong Province (Grant No. 2019WS373).

## Conflict of Interest

Authors C-CH and J-XX were employed by Beijing Deepwise & League of PHD Technology Co., Ltd.

The remaining authors declare that the research was conducted in the absence of any commercial or financial relationships that could be construed as a potential conflict of interest.

## Publisher’s Note

All claims expressed in this article are solely those of the authors and do not necessarily represent those of their affiliated organizations, or those of the publisher, the editors and the reviewers. Any product that may be evaluated in this article, or claim that may be made by its manufacturer, is not guaranteed or endorsed by the publisher.
