# Supplementary material for: Deep Learning Radiomics Nomogram to Predict Lung Metastasis in Soft-Tissue Sarcoma: A Multi-Center Study
Source: Front Oncol. 2022 Jun 24;12:897676. doi: 10.3389/fonc.2022.897676 (PMC9265249; doi:10.3389/fonc.2022.897676)
Supplement: Supplementary file 1 [file DataSheet_1.docx]

**Supplementary material**

**Supplementary item A1: Inclusion and exclusion criteria for the patients**

The inclusion criteria were as follows: 1) each patient had complete medical data; and 2) MRI examination was undertaken within the 2 weeks previous to the clinical intervention. The exclusion criteria were as follows: 1) undergoing clinical intervention before the MRI examination; 2) patients with incomplete medical and/or imaging data (lack of chest CT imaging); 3) patients with low quality images (signal-to-noise ratio < 1.0); 4) patients with other malignant oncology before treatment; and 5) any reason resulting in loss to follow-up.

**Supplementary item A2: MRI acquisition**

The MRI scanners included: GE HDx 1.5-T and 3.0-T (GE Medical Systems), Siemens 3.0-T Magnetom Skyra and Prisma (Siemens Healthineers), and Philips Achieva 1.5-T (Philips Healthcare). The MRI scanning parameters were set as follows: T1WI, 500–600 ms repetition time (TR), 10–15 ms echo time (TE); FS-T2WI 2400–4500 ms TR, 70–120 ms TE. The two sequences had similar 3–5 mm slice thickness, 1 mm slice spacing, 320 × 320 matrix and 200–400 mm field of view.

**Supplementary item A3: MRI semantic feature evaluation**

The MRI semantic features were evaluated by three radiologists who had been engaged in musculoskeletal system diagnosis for more than 3 years. The semantic features included: (1) number (solitary or multiple); (2) depth (deep or superficial, maximum lesion depth ≥ 8 cm was defined as deep); (3) heterogeneous signal intensity on T1WI (< 50% or ≥ 50%); (4) heterogeneous signal intensity on FS-T2WI (< 50% or ≥ 50%); (5) tumor volume showing necrotic MRI signal (none, 1%–50% or ≥ 50% of tumor volume); (6) peritumoral edema (without, with limited, or with extensive peritumoral edema); and (7) location (limb, trunk wall, head and neck, or internal trunk wall). The semantic features were acquired from the largest area of the lesion. When a dispute occurred in the feature analysis, the three radiologists reviewed the MR imaging together and reached a consensus decision.

**Supplementary item A4: Handcrafted feature extraction**

One junior musculoskeletal radiologist (S.L.) drew the ROIs twice, on both the FS-T2WI and T1WI. The ROIs were drawn section-by-section around the edge of the target mass. The ROIs contained the entire mass but excluded visible tumor edema. A senior radiologist (H.W.) validated each segmentation. The hand-crafted feature parameters with ICCs > 0.75 were defined as highly stable in the intra-observer and inter-observer consistency tests and were selected for analysis.

This study applied the Dr. Wise Multimodal Research Platform (<https://keyan.deepwise.com,V1.6.2>) (Beijing Deepwise & League of PHD Technology Co., Ltd, Beijing, China) for hand-crafted feature extraction. Overall, 1379 features were derived from each ROI. The features contained first-order features, shape features, global texture features and local texture features. Global texture features and local texture features contained gray level co-occurrence matrix features, gray level size zone matrix features, gray level run length matrix features, and gray level dependence matrix grayscale features.

**Table S1.** Pathologic Results of 242 Soft-tissue Sarcoma Patients

|  | Training set  (N=116) | External validation set (N=126) |
| --- | --- | --- |
| Liposarcoma | 15 | 19 |
| Myxofibrosarcoma | 29 | 19 |
| Synovial sarcoma | 9 | 16 |
| Pleomorphic liposarcoma | 2 | 5 |
| Undifferentiated pleomorphic sarcoma | 14 | 13 |
| Malignant peripheral nerve sheath tumour | 3 | 1 |
| Leiomyosarcoma | 8 | 10 |
| Angiosarcoma | 2 | 5 |
| Rhabdomyosarcoma | 4 | 9 |
| Spindle cell rhabdomyosarcoma | 1 | 3 |
| Extraskeletal osteosarcoma | 6 | 3 |
| Epithelioid sarcoma | 1 | 1 |
| Alveolar soft part sarcoma | 4 | 2 |
| Extraskeletal myxoid chondrosarcoma | 3 | 3 |
| Spindle cell sarcoma, undifferentiated | 3 | 3 |
| Kaposi sarcoma | 1 | 8 |
| Clear cell sarcoma | 1 | 0 |
| Solitary fibrous tumour, malignant | 3 | 0 |
| Undifferentiated sarcoma | 3 | 3 |
| Others | 4 | 3 |

**TABLE S2. The packages applied in R software**

| Step | R package |
| --- | --- |
| Minimum redundancy maximum relevance | mRMRe |
| Least absolute shrinkage and selection operator | glmnet |
| Receiver operating characteristics curve | pROC |
| Nomogram and calibration curve analysis | Hmisc, lattice, survival, Formula, ggplot2, rms |
| Decision curve analysis | rmda |

**Table S3.** Input Features of Predictive Models

| HD-Combined model | T2DFs_292  T2DFs_286  T2DFs_193  T1DFs_434  T2_log.sigma.3.0.mm.3D_glcm_ClusterProminence  T2DFs_291 |
| --- | --- |
| HCR model | T2_exponential_ngtdm_Busyness  T1_wavelet.HH_firstorder_Maximum  T1_logarithm_glcm_Imc2  T2_wavelet.LL_firstorder_Kurtosis  T1_log.sigma.5.0.mm.3D_ngtdm_Busyness  T1_log.sigma.5.0.mm.3D_glcm_Imc2  T2_lbp.2D_gldm_DependenceNonUniformityNormalized  T1_square_glcm_Correlation  T1_gradient_glszm_SmallAreaLowGrayLevelEmphasis  T1_logarithm_glcm_ClusterShade  T2_exponential_glrlm_ShortRunLowGrayLevelEmphasis  T1_lbp.2D_gldm_LargeDependenceHighGrayLevelEmphasis  T2_log.sigma.3.0.mm.3D_glcm_ClusterProminence |
| DL model | T2DFs_497  T1DFs_311  T2DFs_193  T2DFs_286  T2DFs_160  T1DFs_140  T2DFs_291 |

**TABLE S4.** Results of Radiomics Signature Predictive Performance

| Radiomics signatures | | | | | | | | |
| --- | --- | --- | --- | --- | --- | --- | --- | --- |
| Signature | Classifier | Set | AUC (95%CI) | ACC | SEN | SPE | PPV | NPV |
| HD-Combined signature | LR | Training | 0.928(0.867-0.989) | 0.922 | 0.650 | 0.979 | 0.867 | 0.931 |
|  |  | External validation | 0.791(0.665-0.917) | 0.881 | 0.368 | 0.972 | 0.700 | 0.897 |
|  | DT | Training | 0.790(0.679-0.901) | 0.914 | 0.600 | 0.979 | 0.957 | 0.922 |
|  |  | External validation | 0.758(0.643-0.874) | 0.921 | 0.526 | 0.991 | 0.909 | 0.922 |
|  | RF | Training | 1.000(1.000-1.000) | 0.991 | 0.950 | 1.000 | 1.000 | 0.990 |
|  |  | External validation | 0.791(0.665-0.917) | 0.889 | 0.316 | 0.996 | 0.857 | 0.891 |
|  | SVM | Training | 0.914(0.839-0.988) | 0.828 | 0.000 | 1.000 | NA | 0.828 |
|  |  | External validation | 0.806(0.682-0.930) | 0.849 | 0.000 | 1.000 | NA | 0.849 |
|  | Ada-boost | Training | 0.996(0.990-1.000) | 0.966 | 0.850 | 0.990 | 0.944 | 0.969 |
|  |  | External validation | 0.733(0.593-0.873) | 0.841 | 0.368 | 0.925 | 0.467 | 0.892 |
| HCR signature | LR | Training | 0.856(0.773-0.939) | 0.862 | 0.400 | 0.958 | 0.667 | 0.885 |
|  |  | External validation | 0.590(0.435-0.745) | 0.833 | 0.263 | 0.935 | 0.417 | 0.877 |
|  | DT | Training | 0.917(0.858-0.977) | 0.888 | 0.600 | 0.948 | 0.706 | 0.919 |
|  |  | External validation | 0.550(0.411-0.689) | 0.762 | 0.105 | 0.879 | 0.133 | 0.847 |
|  | RF | Training | 0.999(0.998-1.000) | 0.991 | 0.950 | 1.000 | 1.000 | 0.990 |
|  |  | External validation | 0.590(0.435-0.745) | 0.825 | 0.105 | 0.953 | 0.286 | 0.857 |
|  | SVM | Training | 0.808(0.690-0.926) | 0.836 | 0.050 | 1.000 | 1.000 | 0.835 |
|  |  | External validation | 0.600(0.444-0.756) | 0.857 | 0.053 | 1.000 | 1.000 | 0.856 |
|  | Ada-boost | Training | 1.000(1.000-1.000) | 1.000 | 1.000 | 1.000 | 1.000 | 1.000 |
|  |  | External validation | 0.560(0.422-0.697) | 0.762 | 0.158 | 0.869 | 0.176 | 0.853 |
| DL signature | LR | Training | 0.909(0.824-0.994) | 0.922 | 0.600 | 0.990 | 0.923 | 0.922 |
|  |  | External validation | 0.771(0.636-0.906) | 0.905 | 0.474 | 0.981 | 0.818 | 0.913 |
|  | DT | Training | 0.784(0.673-0.896) | 0.905 | 0.600 | 0.969 | 0.800 | 0.921 |
|  |  | External validation | 0.749(0.633-0.866) | 0.905 | 0.526 | 0.972 | 0.769 | 0.920 |
|  | RF | Training | 1.000(1.000-1.000) | 1.000 | 1.000 | 1.000 | 1.000 | 1.000 |
|  |  | External validation | 0.771(0.636-0.906) | 0.873 | 0.316 | 0.972 | 0.667 | 0.889 |
|  | SVM | Training | 0.892(0.795-0.989) | 0.889 | 0.400 | 1.000 | 1.000 | 0.889 |
|  |  | External validation | 0.773(0.640-0.905) | 0.881 | 0.211 | 1.000 | 1.000 | 0.877 |
|  | Ada-boost | Training | 0.991(0.973-1.000) | 0.974 | 0.850 | 1.000 | 1.000 | 0.970 |
|  |  | External validation | 0.753(0.612-0.893) | 0.905 | 0.474 | 0.981 | 0.818 | 0.913 |

Note: AUC: area under the receiver operating characteristic curve; CI: confidence interval; LR: logistic regression; DT: decision tree; RF: random forest; SVM: support vector machine; Ada-boost: adaptive boosting; NA: not available; ACC: accuracy; SEN: sensitivity; SPE: specificity; PPV: positive predictive value; NPV: negative predictive value

**TABLE S5.** Results of Radiomics Signature with the Synthetic Minority Oversampling Technique Predictive Performance

| Radiomics signatures with SMOTE | | | | | | | | |
| --- | --- | --- | --- | --- | --- | --- | --- | --- |
| Signature | Classifier | Set | AUC (95%CI) | ACC | SEN | SPE | PPV | NPV |
| HD-Combined signature | LR | Training | 0.939(0.908-0.970) | 0.835 | 0.788 | 0.875 | 0.840 | 0.832 |
|  |  | External validation | 0.776(0.653-0.898) | 0.778 | 0.474 | 0.832 | 0.333 | 0.899 |
|  | DT | Training | 0.829(0.778-0.881) | 0.818 | 0.950 | 0.708 | 0.731 | 0.944 |
|  |  | External validation | 0.629(0.508-0.749) | 0.627 | 0.632 | 0.626 | 0.231 | 0.905 |
|  | RF | Training | 1.000(1.000-1.000) | 1.000 | 1.000 | 1.000 | 1.000 | 1.000 |
|  |  | External validation | 0.776(0.653-0.898) | 0.817 | 0.579 | 0.860 | 0.423 | 0.920 |
|  | SVM | Training | 0.914(0.876-0.953) | 0.755 | 0.463 | 1.000 | 1.000 | 0.691 |
|  |  | External validation | 0.799(0.675-0.922) | 0.881 | 0.263 | 0.991 | 0.833 | 0.883 |
|  | Ada-boost | Training | 0.997(0.993-1.000) | 0.977 | 0.963 | 0.990 | 0.987 | 0.969 |
|  |  | External validation | 0.778(0.634-0.923) | 0.841 | 0.632 | 0.879 | 0.480 | 0.931 |
| HCR signature | LR | Training | 0.877(0.825-0.929) | 0.795 | 0.813 | 0.781 | 0.756 | 0.833 |
|  |  | External validation | 0.558(0.401-0.714) | 0.675 | 0.316 | 0.738 | 0.176 | 0.859 |
|  | DT | Training | 0.959(0.932-0.986) | 0.909 | 0.875 | 0.938 | 0.921 | 0.900 |
|  |  | External validation | 0.479(0.346-0.611) | 0.714 | 0.211 | 0.804 | 0.160 | 0.851 |
|  | RF | Training | 1.000(1.000-1.000) | 1.000 | 1.000 | 1.000 | 1.000 | 1.000 |
|  |  | External validation | 0.588(0.401-0.714) | 0.722 | 0.211 | 0.813 | 0.167 | 0.853 |
|  | SVM | Training | 0.972(0.952-0.992) | 0.909 | 0.925 | 0.896 | 0.881 | 0.935 |
|  |  | External validation | 0.575(0.420-0.729) | 0.738 | 0.316 | 0.813 | 0.231 | 0.870 |
|  | Ada-boost | Training | 0.973(0.951-0.996) | 0.943 | 0.950 | 0.938 | 0.927 | 0.957 |
|  |  | External validation | 0.432(0.299-0.566) | 0.706 | 0.368 | 0.766 | 0.219 | 0.872 |
| DL signature | LR | Training | 0.927(0.888-0.966) | 0.847 | 0.825 | 0.865 | 0.835 | 0.856 |
|  |  | External validation | 0.758(0.618-0.898) | 0.794 | 0.579 | 0.832 | 0.379 | 0.918 |
|  | DT | Training | 0.932(0.893-0.972) | 0.915 | 0.838 | 0.979 | 0.971 | 0.879 |
|  |  | External validation | 0.683(0.547-0.820) | 0.746 | 0.526 | 0.785 | 0.303 | 0.903 |
|  | RF | Training | 1.000(1.000-1.000) | 1.000 | 1.000 | 1.000 | 1.000 | 1.000 |
|  |  | External validation | 0.758(0.618-0.898) | 0.770 | 0.211 | 0.869 | 0.222 | 0.861 |
|  | SVM | Training | 0.912(0.869-0.956) | 0.784 | 0.600 | 0.938 | 0.889 | 0.738 |
|  |  | External validation | 0.776(0.643-0.909) | 0.889 | 0.421 | 0.972 | 0.727 | 0.904 |
|  | Ada-boost | Training | 0.987(0.976-0.997) | 0.932 | 0.963 | 0.906 | 0.895 | 0.967 |
|  |  | External validation | 0.702(0.563-0.842) | 0.817 | 0.579 | 0.860 | 0.423 | 0.920 |

Note: SMOTE: synthetic minority oversampling technique; AUC: area under the receiver operating characteristic curve; CI: confidence interval; LR: logistic regression; DT: decision tree; RF: random forest; SVM: support vector machine; Ada-boost: adaptive boosting; ACC: accuracy; SEN: sensitivity, SPE: specificity; PPV: positive predictive value; NPV: negative predictive value


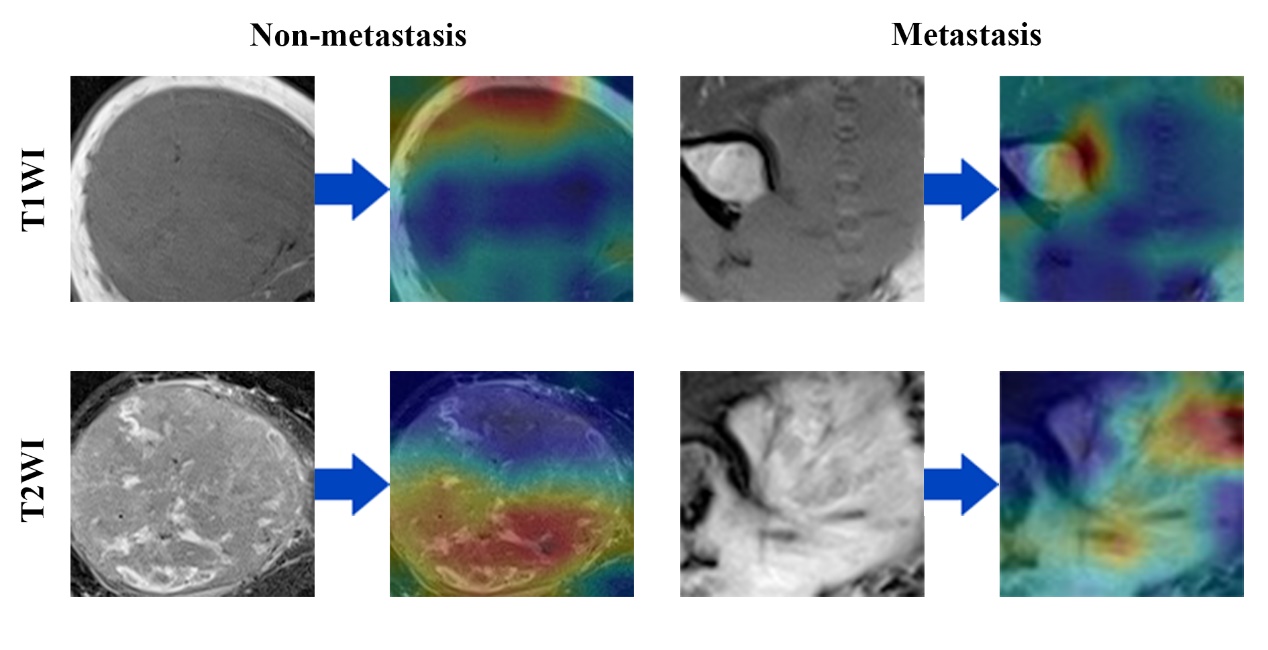


**Figure S1.** Activation maps of the deep convolutional neural networks for soft tissue sarcoma lung metastasis vs. non-metastasis (reflecting the important areas associated with the risk of lung metastasis) were obtained from the T1WI and FS-T2WI. The highlighted areas represent the regions of high value in the model’s lung metastasis prediction, whereas the suppressed areas are regions with little important predictive value for lung metastasis.
